# Development of the palliative care referral system: proposal of a tool for the referral of cancer patients to specialized palliative care

**DOI:** 10.1186/s12904-022-01094-0

**Published:** 2022-11-28

**Authors:** Alessandra Pigni, Sara Alfieri, Augusto Tommaso Caraceni, Ernesto Zecca, Viviana Fusetti, Antonino Tallarita, Cinzia Brunelli

**Affiliations:** 1 Palliative care, Pain Therapy and Rehabilitation Unit, Fondazione IRCCS Istituto Nazionale dei Tumori, Milan, Italy; 2 Clinical Psychology Unit, Fondazione IRCCS Istituto Nazionale dei Tumori, Milan, Italy; 3 Dipartimento di Scienze Cliniche e di Comunità, Università degli Studi di Milano, Milan, Italy; 4 Università degli Studi di Roma “Tor Vergata”, Rome, Italy

**Keywords:** Palliative care, Neoplasm, Referral, Consultation, Nominal group technique

## Abstract

**Background:**

Early palliative care (PC) has shown beneficial effects for advanced cancer patients. However, it is still debated what criteria to use to identify patients for PC referral.

**Aim:**

To document the initial steps of the development of the Palliative Care Referral System (PCRS), a tool to be used by oncologists in clinical practice.

**Methods:**

A multiprofessional working group developed the PCRS based on the results of a scoping literature review on PC referral criteria. PCRS criteria were evaluated by experts via a nominal group technique (NGT). Descriptive statistics were used to summarize expert scores on relevance, appropriateness and perceived feasibility of the criteria proposed. Quotations of participants during the discussion were also reported.

**Results:**

Sixteen studies, including PC referral criteria/tools, emerged from the scoping review. Severe symptoms, poor performance status, comorbidities and prognosis were the most commonly used criteria. The PCRS included nine major criteria and nine assessment methods; a scoring procedure was also proposed. Answers to the questionnaire during the NGT showed that five criteria reached full agreement on all items, while four did not, and were then discussed within the group. Participants agreed on the relevance of all criteria and on the appropriateness of methods proposed to assess most of them, while issues were raised about potential feasibility of the overall assessment of the PCRS in clinical practice.

**Conclusion:**

The PCRS has been developed as an help for oncologists to timely identify patients for specialized PC referral. Since feasibility emerged as the main concern, implementation strategies have to be tested in subsequent studies.

## Introduction

The World Health Organization defines palliative care (PC) “an approach that improves the quality of life of patients and their families by means of early identification and impeccable assessment and treatment of pain and other problems, physical, psychosocial and spiritual” [1]. This definition already underlines that patients should have access to PC as soon as they need in the course of an incurable disease.

Patients with advanced cancer have historically been the first to benefit from PC, but although PC has been part of comprehensive patient care for decades when the disease no longer responds to therapies with curative intent [2], the timing and criteria for referring patients to PC at a time are still poorly defined.

More specifically, while randomized controlled trials (RCT) have shown that early referral to specialized PC improves patients’ and care givers’ quality of life in advanced cancer care [3–6], they do not translate in individualized indication to PC in clinical practice.

In fact, in most RCT, listed in the Cochrane review published in 2017 the timing for referral to PC was set within 8 weeks from the diagnosis of advanced disease [7]. The same timing has been then suggested by the American Society of Clinical Oncology to define early palliative care [8].

However, this is a relatively generic definition to be applied to all patients in all settings, it may result in overloading limited PC resources and is therefore of limited practical use. One approach could be to identify criteria that can be shared between oncologists and palliative care specialists based on patients needs and clinical conditions [9].

In 2016, a systematic literature review was published by Hui et al. which identified 20 criteria, including 6 recurrent themes for cancer outpatient PC referral. This review highlights the significant heterogeneity regarding the timing and process for referral and the need for further research to develop standardized criteria [10].

Of the proposed screening tools for PC referral, some have been developed in general medical practice and are not specific enough to be used in cancer care (as for example NECPAL e SPICT) [11, 12]. More specific criteria for oncology have also been proposed, but none is widely used and their impact and utility have yet to be proven [13].

The aim of the present work is to document the initial phases of the development process of the Palliative Care Referral System (PCRS), for patients with advanced cancer, based on published evidences combined with consensus between oncologists and PC specialists. In this initial step our goal was to obtain a tool applicable to oncological clinical practice. Field testing and implementation of the PCRS will follow in subsequent studies to test its impact on user perception of quality of care received, quality of life and the use of healthcare resources [14].

## Material and method

The study was carried out in three phases: 1) identification and mapping of operational criteria used to refer advanced cancer patients to timely PC, as reported in the literature; 2) development of the first version of the PCRS based on the above criteria; 3) consensus assessment about relevance, appropriateness and feasibility of PCRS implementation in routine clinical practice, by oncologists and palliative care specialists and design of the PCRS version to be used in the implementation study. By feasibility, in the present paper it is meant the experts’ perception of potential feasibility of the tool implementation in clinical practice.

### Phase 1: identification and mapping of published PC referral criteria

A scoping literature review was performed, searching PubMed from Jan 2014 to Dec 2020 to integrate a previous systematic review [10]; hand searching of retrieved papers was performed. The search strategy, reported in Table 1, used the terms “cancer”, “referral”, “needs”, “palliative care “ and “supportive care”. Eligible papers had to include standardized PC referral criteria or tools in patients with advanced cancer. No language or study design limits were introduced. Three researchers reviewed abstract for eligibility evaluation and identified relevant papers.

**Table 1:** Search strategy applied in Medline

Search number	Query	Filters	Results
5	#1 AND #2 AND #3	from 2014 to 2020	3241
4	#1 AND #2 AND #3		5936
3	(palliative care) OR (“supportive care”)		97,689
2	referral [tw] or needs [tw]		534,738
1	cancer [tiab] OR neoplasms [mh] OR tumour [tiab] OR oncol*[tiab] OR carcinoma*[tiab] OR malignan*[tiab]		4,129,517

### Phase 2: development of the first version of the PCRS

Based on the analysis of the macro-areas for PC referral criteria in selected papers, three researchers with PC expertise identified the domains most frequently used and prepared a list of indicators.

This list was discussed within a research team composed by a multiprofessional working group (one oncologist, 2 PC specialists, one nurse, one psychologist, one assessment methods expert) to identify the most clinically relevant criteria, the most suitable measurement tools and the most suitable professional (physician, nurse, case manager). to appoint to data collection. As a result, a first version of the PCRS including, for each chosen criterion, assessment methods and scoring procedure was submitted to consensus evaluation among clinicians.

### Phase 3: consensus assessment

In order to understand if the criteria identified were shared by oncologists and PC specialists, a Nominal group technique (NGT) was used. Compared to other kinds of qualitative group research (e.g. focus group), NGT is a methodological approach designed to build consensus [15] and, for this reason, it was deemed appropriate for our research objectives. According to NGT, a group of people who are considered “experts” with respect to a topic or a situation takes part in a structured facilitated discussion [16] aimed at reaching a consensus about a topic. Unlike what happens in a Delphi consensus, where the experts do not meet and do not know each other, in the NGT a “face to face” confrontation is elicited, despite the vote being anonymous. As all the professionals involved know each other and agree with the objective of co-constructing a tool, it is essential that all the professionals involved openly confront each other.

### Participants

Experts involved in the NGT session were the referring physicians of the five outpatient oncology clinics (gastro-enteric, head&neck, genitourinary, lung cancer and sarcoma) participating in the implementation of the PCRS that is now ongoing [14], as well as the PC specialists working at the outpatient PC clinic of our Center. The choice of those five oncology clinics was aimed at covering cancer diseases with heterogeneous prevalence and was also determined by the interest of the involved teams to participate in the research.

### Procedures

The NGT session was moderated by a psychologist, expert in qualitative research [AS], and it was conducted online due to the restrictions imposed by the Covid-19 pandemic; the session lasted about 3 and a half hours; it was videotaped and verbatim transcribed, with the consent of the participants.

The NGT session was implemented according to the following steps:

- STEP A: presentation to the participants of the PCRS by a researcher experienced in palliative care [EZ]. For each of the identified criteria the presentation addressed:
  - ◦ description of the criterion and relevance for PC referral;
  - ◦ description of the method of assessment;
  - ◦ professionals appointed to the assessment (eg. physician or nurse).
- STEP B: silent, individual responses of participants to an online questionnaire about relevance of the criteria (related to specific cancer disease), appropriateness (applied to the assessment methods) and perceived feasibility in clinical practice, for each criterion.
- STEP C: scoring of the online questionnaire and presentation of results to the participants by the moderator.
- STEP D: group discussion of items where consensus was not reached.
- STEP E: list of aspects on which consensus has been reached and on which it has not been reached.
- STEP F: decision about final tool for implementation.

Two types of data are obtained from the NGT: quantitative data (answers to the online questionnaire) and qualitative data (quotations of the participants about the reasons in support or against an aspect considered).

Quantitative data: For each criterion in the PCRS, participants scored on a 4-step Likert scale (from 1 = not at all to 4 = a lot) the following items: a) relevance of the PC referral criterion; b) appropriateness of the measurement tool proposed for assessment; c) perceived feasibility of the assessment in routine clinical practice. Expert responses were considered to be in a “positive agreement” when all respondents provided scores of 3 or 4, otherwise they were considered “disagreement”.

Qualitative data: Some of the most interesting quotations on disagreement expressed by participants during discussion have been reported in the “results” section.

## Results

### Phase 1: literature review of PC referral criteria

Three thousand, two hundred forty-one papers were initially retrieved from the MEDLINE search, and 77 full text articles were examined; 11 papers were added from hand search. From the 88 paper examined in full text, 16 papers [17–33], were chosen because they used tools to identify criteria for PC referral. The tools were quite heterogeneous both in length (from 7 to 58 items) and in the criteria adopted. The most frequently addressed dimensions were: uncontrolled symptoms (16/16), poor performance status (10/16), communication and decision making issues (9/10), prognosis (8/16) and comorbidities (6/16) (Table 2). In 13/16 studies data were reported by physicians or nurses, in 6/16 tools were prospectively applied in clinical practice and 4/16 tools included patient reported outcomes (data not reported in table).

**Table 2:** list of papers selected and palliative care referral dimensions used

Author/Year	Tools	Dimensions							
		Advanced disease	Prognosis	Performance Status	Symptoms	Communication/ Decision making	Comorbidities	Blood values	Other
[1] Carrasco-Zafra, 2020	PPS; IDC-Pal			X	X		X		functional autonomy; family support
[2] DiLello, 2018	palliative care screening tool (PCST)	X	X	X	X		X		functional autonomy
[3] Gemmell, 2020	7 different tools	X	X	X	X		X		psychosocial problem
[4] Glare, 2015	11 item PC screening tool	X	X	X	X	X	X		patient/family requests PC consult, prolonged length of stay, moderate-severe distress
[5] Goldberg, 2016	7 item “Living with cancer” PRO screening tool			X	X				financial and family burden
[6] Hui, 2020	9 needs-based criteria and 2 time-based criteria	X	X		X	X			brain metastases; delirium, spinal cord compression, request for hastened death, patient requests PC consult, emotional symptoms, spiritual or existential crisis
[7] Le, 2020	11-item screening tool	X	X	X	X	X	X		patient/family requests PC consult prolonged length of stay, moderate-severe distress
[8] Molin, 2019	PALLIA-10 questionnaire	X	X	X	X	X		X	psychological and social factors, patient/family requests PC consult, patient requests euthanasia
[9] Ohno, 2017	PPI-Kripp’s model	X	X	X	X			X	delirium
[10] Ostgathe, 2019	10 item PC tool	X		X	X	X	X		brain metastases, delirium, patient/family requests PC consult, distress, prolonged length of stay
[11] Paiva, 2020	16 item PC referral protocol	X		X	X	X			brain metastasis, delirium, patients requests PC consult, severe emotional symptoms, suicide risk
[12] Rauenzahn, 2017	10 item ESAS				X				
[13] Sterie, 2020	17 item IPOS				X	X			psychological items, functional autonomy
[14] Ullgren, 2017	EORTC QLQ C-30 - INFO 25 (58 items tool)		X		X				financial items, emotional items, psychological items
[15] Yun, 2018	32 item Quality Care Questionnaire PC				X	X			psychological, social and spiritual support
[16] Zeneli, 2016	Supportive Care Needs Survey-SF 34 items				X	X			psychological needs, sexuality

*Legenda: PC* Palliative care, *PPS* Palliative performance scale, *IDC* Diagnostic Instrument for Complexity in Palliative Care, *PPI* Palliative Prognostic Index, *ESAS* Edmonton Symptom Assessment System, *IPOS* Integrated Palliative care Outcome Scale, *EORTC* European Organization for Research and Treatment of Cancer questionnaire, *SF* Short Form

### Phase 2: development of the first version of the PCRS

By processing the criteria highlighted by the selected studies and in the previous literature review [10] a first list of 19 areas relevant for PC referral was drawn up and then reduced to a final list of nine non overlapping criteria (Table 3) to be submitted to expert clinicians discussion.

**Table 3:** List of criteria and related score for the development of an outpatients palliative care referral tool

CRITERIA	MEASURE/TOOL	CUT OFF/SCORING	EVALUATOR	MAXIMUM SCORE
Performance Status	ECOG	0 = 0 points 1 = 1 point 2 = 2 points 3 = 3 points 4 = 4 points	Physician during visit	**4**
Prognosys	Clinical evaluation	0-3 moths = 2 points 3-12 months = 1 point > 12 months = 0 points	Physician during visit	**2**
Decision making/ Communication issue	when the oncologist has to inform the patient about: change of therapy, discontinuation of active treatment	change of therapy = 1 point discontinuation of active treatment = 2 point	Physician during visit	**2**
Physical symptoms	ESAS (11 items; no anxiety and depression)	0.5 points each symptom≥5	Nurse/case manager before visit	**5.5**
Psychological symptoms	ESAS (anxiety and depression only)	at least one ≥5 = 1 point	Nurse/case manager before visit	**1**
Social Problems	Items from Distress Termometre	at least two = 1 point	Nurse/case manager before visit	**1**
Serious Comorbidities	Charlson comorbidity Items (but tumour)	None = 0 points One or Two = 1 point >Two = 2 points	Team (before and/or during visit)	**2**
Other Relevant Conditions	Clinical evaluation		Team (before and/or during visit)	**6**
	Brain metastase	Yes = 1 point No = 0 points		
	Bone metastases	Yes = 1 point No = 0 points		
	Abnormal blood levels^a^	Yes = 1 point No = 0 points		
	Neoplastic effusion	Yes = 1 point No = 0 points		
	Cognitive impairment/ delirium	Yes = 1 point No = 0 points		
	Other syndrome or complication due to cancer	Yes = 1 point No = 0 points		
Patient or caregiver request	Patient or caregiver require access to palliative care	Yes = 1 point No = 0 points	Team (before and/or during visit)	**1**
				**24.5**

*Legenda: ESAS* Edmonton Symptom Assessment System

^a^ = Anaemia, high Creatininemia, high Bilirubinemia, high Calcemia and low Albuminemia level

### Phase 3: consensus assessment of the proposed tool and design of PCRS version to be used in the implementation study

Nine professionals attended the meeting, six and three respectively working in the oncology and palliative care outpatient clinics. Eight of them were female; the mean age was 38.28 years (range: 32-49; SD = 6.09) and the mean time since working with cancer patients was 5.25 years (range: 1-17; SD = 5.09).

#### Individual participant quantitative scoring

Based on the participant scoring of the PCRS items, five criteria (Performance Status, Decision making/communication issues, Social Problems, Serious Comorbidities, Request for sending to PC by the patient or a family member), reached complete agreement on all the items considered (relevance appropriateness and feasibility) while four did not (Prognosis, Physical Symptoms, Psychological Symptoms, Other relevant conditions); therefore, the discussion focused on the latter items.

#### Group discussion

Regarding psychological symptoms, the relevance of the criterion resulted to be generally endorsed: *“The psychological aspect is often a symptom of the disease, which in any case affects the patient’s experiences, the tolerability of treatments, can also change the objectives of the treatments ... I use to tell patients that if they don’t put their head, it is useless that we give the medicines […] also because the weight of the physical symptoms depends on the psychological ones, too”* (participant n°1-p1). All the participants agreed with the expressed position, some even hypothesized an error in completing the questionnaire: *“I think we are all in agreement with what my colleague has masterfully expounded before ... I don’t see how we could disagree […] Maybe someone was wrong to answer ...”* (p2). With respect to the appropriateness of the ESAS used for the detection of psychological symptoms and assessment feasibility, oncologists declare not to be qualified to judge the goodness of an instrument to assess psychological aspects: *“... I don’t think I have the skills, I don’t know what are the tools that are used to evaluate the psychology of a patient, because I am not a psychologist, I am an oncologist, I get excited when I see the survival curves[…] I rely on empathy ... […] on the relationship with the patient. […] Other tools are useful in an ideal world ... in my clinic where I am forced to make visits last in a short time, because there is a kilometer-long list of patients waiting ... […]I cannot, I am unable to do this evaluation.”* (p3). Furthermore, they do not perceive the detection of psychological symptoms before the visit as feasible, mostly they fear that different tools/professionals may evaluate aspects that are not “objective” in different ways: *“one thing that worries me a little is having information in the medical record that does not always agree... […]these patients are also in clinical trials, they do quality of life questionnaires in which they answer these questions in another way and frankly I am also afraid let the patient tell me: 'how many times do you have to ask me the same things in different ways?'”* (p2).

About physical symptoms, there is agreement with respect to the relevance, but problems emerge concerning tool and feasibility. “*If I think of my practice where [...] the visit lasts only 5 minutes, even looking at the questionnaire becomes very complicated”* (p4) *“you have to go to another page [of the electronic medical record], fill in other information and send it, which seems trivial ... but with the times that oncologists have, this is tiring too”*(p5). Furthermore, only one participant states that she is familiar with ESAS and that she used it in the past, pointing out problems of understanding of the items by patients and, therefore, of interpretation of scores by professionals.

About prognosis, the critical aspect that emerges is related to the appropriateness of the “prediction” criteria. Actually, oncologists reveal to be concerned to make an incorrect prediction of the patient’s prognosis: *“it is extremely difficult to be able to give a survival cut-off of 3, 6 or 12 months. The relevance of this criterion is unquestionable ... but the cut off is a bit random ...”* (p6). After extensive discussion, focused on the fact that every oncologist is likely to have an idea (albeit approximate) of a patient’s survival and that this tool should not be seen as a “death sentence” for the patient, one oncologist states that *“I am convinced by the colleague’s explanations. What I would like to emphasize is that this is an absolutely subjective clinical parameter, so if we take three oncologists who have to do the evaluation on the same patient, we will have three different judgments”* (p2). The fear of “subjectivity” therefore seems to persist. In this regard, the intervention of a PC professional is decisive: *“we have patients here in the clinic that we have been following for 6 years, so this is not the problem. […] What we regret is when the patient arrives too late to handle it ... […] Don’t worry about your clinical impression, no one will tell you “that doctor made a mistake ... the patient lived longer ...“ welcome patients to live longer!* “ (p7). The discussion then moves on to feasibility: *“the colleague is right, I think this tool sees the things that need to be seen, the problem is just understanding how to do it, because we already don’t know where to find the time to do it. Every extra element is an effort. So my perplexity is to find the way and the time to do things, because otherwise I risk not to do them”* (p2). All participants seem to agree with this view.

As regard to “other relevant conditions”, the discussion focuses on the relevance of blood value alterations - because all the participants consider neoplastic effusions and cognitive alterations/ delirium to be relevant - and once again suggest the hypothesis that someone has made a mistake in answering. Only one person, on the other hand, declares his perplexity with respect to abnormal blood values: *“I put “little“ in the blood tests thinking that many alterations are induced by our treatments, so it was a way to say that they must be relativized, because they are not necessarily linked to a worsening of the disease.”* (p7). The discussion therefore focuses on the importance of the doctor’s assessment of abnormal blood values, an aspect that reassures the participants and unifies the positions: “*This is true, in terms of relative weight it makes a lot of sense, thanks ...*” (p7). Once again, the participants highlight the difficulty of adding elements to their clinical assessment.

To reinforce the positions previously expressed, some concluding reflections of the group are reported: *“All criteria are important, in an ideal world they should be applied, the problem is that we are in the real world. We have to find a solution to apply these criteria and at the moment I can’t find it. […] I wonder if it is not better to create a parallel clinic, because during the visits, with the waiting lists we have, it is complicated for us. […] Our prayer is to go on feasibility, on practice. We have an average of 18-20 people in the morning and another 9-10 in the afternoon... Especially those in the morning are patients who are inside the protocols, they are not well, they require an incredible bureaucratic and clinical burden, so the morning clinics finish at 3.00 pm. We can also be very motivated, we like this tool, but we have to find a way to apply it to clinical practice ... During the visit we can’t fill in the fields of the medical record, we have to find another way “* (p3).

In addition to the criticisms, there are also interesting suggestions, such as the following: *“I have a comment and a suggestion. […] the reason why the medical record does not work as we all would like is the way it was developed […] in a vertical way and the amount of data, often redundant on every single patient, is unimaginable. We are talking about inserting other fields in the medical record, but they are data that any technician could obtain from the outpatient letter if he put his head in it. The suggestion, even if I don’t know how feasible it is, is to entrust the patient at home with part of the compilation of the Proms. What if the patient uses an app the day before? Personally I would be happy to know that the next day a patient will come who has this, this and that ... maybe I prepare and manage it even better. And this can save a lot of the compilation work”* (p7).

Table 4 summarised the results of NGT in term of agreement and disagreement.

**Table 4:**
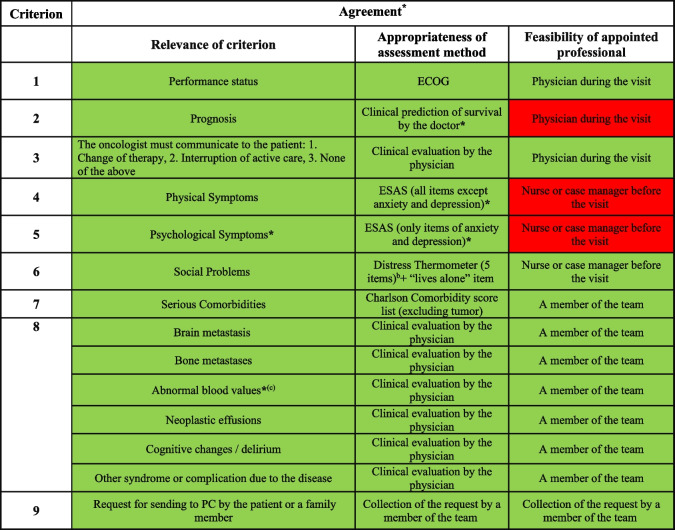
Agreement or disagreement for each criterion proposed

*Legenda:* Green/Y = Yes, positive agreement reached; Red/N = No, positive agreement not reached; ^a^ only for issues where no agreement was reached on the online survey; ^b^ Family Problems: dealing with children; Practical Problems: Housing, Economic problems, Work/school, Transportation; ^c^ Anaemia, high Creatininemia, high Bilirubinemia, high Calcemia and low Albuminemia level.

NGT closed with the agreement among participants to start an implementation study [14] of the tool shown in Table 3 in clinical practice, despite some doubts on overall feasibility.

## Discussion

This study allowed the design of the PCRS, a new screening tool to help oncologists in identifying patients to be referred to PC in their routine practice. Through the scoping review and the comparative analysis of existing tools, the following clinical criteria were included in the PCRS: performance status, prognosis, decision making/communication issues, physical symptoms, psychological symptoms, social problems, serious comorbidities, other relevant conditions and patient or caregiver request. The NGT session, including oncologists and PC physicians, confirmed criteria relevance and appropriateness of assessment measures, while issues were raised about PCRS feasibility in clinical practice (Table 4). The scoping review showed a lack of definition of standardized criteria for PC referral and consequently a quite heterogeneous set of tools proposed; moreover only five of them have been tested in clinical practice [20, 23, 24, 26–28]. As to the criteria included in the screening tools, symptoms, performance status and communication/decision making issues are often considered, while prognosis and comorbidities are less frequent (Table 2). Also during the NGT session in our study, participants raised issues about prognosis as referral criterion but the reasons given to motivate resistance in using it seemed to be dealing more with a difficulty in providing “reliable prognosis estimates” than with actual relevance of the criterion for PC referral. Therefore it was decided to keep it, underlining that it should not be overstated, considering that a multidimensional tool aimed at referring advanced cancer patients to PC cannot disregard such a relevant aspect.

The main difference with the PCRS development in comparison with other already existing tools [20, 21, 23, 24, 26–28], is that none of them have been developed based on a comprehensive literature review and on a structured and formal consensus procedure between oncologists and PC specialists. The PCRS is now being implemented within an ongoing pre-post intervention study [14].

Our final aim is the identification of standardised criteria for referral of outpatients to PC to help oncologists understand the “optimal moment” to integrate cancer care with PC. Currently, in the absence of a validated and established screening and criteria procedure, oncologists refer patients to PC based on perceived needs and their clinical judgement [34]. Late referral to palliative care has been often observed [35–38] and considered one of the factors with a negative impact on the care pathway of patients with advanced cancer [39].

Although the reasons beneath this problem are widely debated among researchers, including palliative care associated stigma and inadequate awareness of PC by oncologists [26], a tool for screening PC needs developed with the participation of oncologists and PC specialists with the aim of subsequent implementation in routine clinical practice could help to bridge this gap and to overcome barriers to timely PC referral.

It is also important that the tool is grounded in the context in which it will be implemented, in order to be more usable for oncologists and therefore more useful for patients. Involving experts in a face-to-face structured meeting enabled first-hand information to be obtained from those working in the “front line” of the clinical areas involved. Capitalizing on the experience and expertise of clinicians in this way underscored our claim that the findings were clinically relevant [40]. NGT makes it clear that the participants agree on the relevance of all the criteria and, generally, also on the proposed tools.

The main problem raised by oncologists, who are the main players in implementing the PCRS in clinical practice, is its feasibility, not particularly for the application of the individual criteria, but for the overall evaluation procedure. They emphasized: lack of time to complete the PCRS during or before the visit, lack of expertise in assessing psychological needs, burden of research related activities which are already implying data collection by clinicians and questionnaire completing by patients [34]. All of this suggests the need to develop an operational strategy to minimize the oncologist’s time in completing the tool.

One possible strategy would be to emphasize the role of nurses and case managers in completing part of the clinical information and in helping the patients to complete the required patient reported outcome measures; nurses and case managers could also participate in a second step in sharing the implementation strategy at the organizational level to make the PCRS a priority and an aid in the clinical process.

This study has limitations. Firstly, the consensus procedure was carried out among a small sample of professionals working in a single cancer center. While this meets our research aim, it may impact generalizability. Secondly the panel of experts for the NGT includes physicians only and therefore it lacks the vision of the other figures involved in the implementation of the PCRS (nurses, care managers, psychologists). We recognise the role and importance of all health professionals in PC, as amply documented in the literature and in the clinical trials that include nurse lead models [3, 41–44]; however our choice to involve oncologists and PC physicians reflects our center clinical practice and the fact that oncologists are accountable for the referral to the PC outpatients service.

## Conclusion

The PCRS was developed to facilitate timely referral of patients to specialized PC by oncologists. Feasibility in routine clinical practices was a main concern by oncologists and implementation strategies involving all stakeholders, namely physicians, nurses psychologists, administrators and also patient advocates, are needed and will be part of subsequent clinical trials.

## Data Availability

The datasets analysed during the current study available from the corresponding author on reasonable request.
